# The Absence of Endothelial Sodium Channel α (αENaC) Reduces Renal Ischemia/Reperfusion Injury

**DOI:** 10.3390/ijms20133132

**Published:** 2019-06-27

**Authors:** Antoine Tarjus, Cecilia González-Rivas, Isabel Amador-Martínez, Benjamin Bonnard, Rebeca López-Marure, Frédéric Jaisser, Jonatan Barrera-Chimal

**Affiliations:** 1 Centre de Recherche des Cordeliers, INSERM UMRS 1138, Sorbonne Université, USPC, Université Paris Descartes, Université Paris Diderot, F-75006 Paris, France; 2 Laboratorio de Fisiología Cardiovascular y Trasplante Renal, Unidad de Medicina Translacional, Instituto de Investigaciones Biomédicas, Universidad Nacional Autónoma de México and Instituto Nacional de Cardiología Ignacio Chávez, Mexico City 04510, Mexico; 3 Departamento de Fisiología, Instituto Nacional de Cardiología Ignacio Chávez, Mexico City 14080, Mexico; 4 INSERM U1116, Clinical Investigation Centre, Lorraine University, 54052 Vandoeuvre-lès-Nancy, France; 5 INI-CRCT (Cardiovascular and Renal Clinical Trialists) F-CRIN Network, 31059 Nancy, France

**Keywords:** ENaC, endothelium, endothelial cell stiffness, acute kidney injury, endothelial nitric oxide synthase

## Abstract

The epithelial sodium channel (ENaC) has a key role in modulating endothelial cell stiffness and this in turn regulates nitric oxide (NO) synthesis. The physiological relevance of endothelial ENaC in pathological conditions where reduced NO bioavailability plays an essential role remains largely unexplored. Renal ischemia/reperfusion (IR) injury is characterized by vasoconstriction and sustained decrease in renal perfusion that is partially explained by a reduction in NO bioavailability. Therefore, we aimed to explore if an endothelial ENaC deficiency has an impact on the severity of renal injury induced by IR. Male mice with a specific endothelial sodium channel α (αENaC) subunit gene inactivation in the endothelium (endo-αENaC^KO^) and control littermates were subjected to bilateral renal ischemia of 22 min and were studied after 24 h of reperfusion. In control littermates, renal ischemia induced an increase in plasma creatinine and urea, augmented the kidney injury molecule-1 (Kim-1) and neutrophil gelatinase associated lipocalin-2 (NGAL) mRNA levels, and produced severe tubular injury. The absence of endothelial αENaC expression prevented renal tubular injury and renal dysfunction. Moreover, endo-αENaC^KO^ mice recovered faster from renal hypoxia after the ischemia episode as compared to littermates. In human endothelial cells, pharmacological ENaC inhibition promoted endothelial nitric oxide synthase (eNOS) coupling and activation. Altogether, these data suggest an important role for endothelial αENaC in kidney IR injury through improving eNOS activation and kidney perfusion, thus, preventing ischemic injury.

## 1. Introduction

Acute kidney injury (AKI) induced by ischemia/reperfusion (IR) is a major clinical concern that affects around 10% of hospitalized patients and up to 40–60% of critical care patients [1,2]. The physiopathology of AKI is not completely understood and there is a lack of therapeutic agents to treat AKI in daily clinical practice [3]. A hallmark of renal IR is the prolonged reduction in the renal blood flow and oxygen supply to the kidney [4,5,6]. The reduction in the kidney perfusion is triggered by vasoconstriction due to an imbalance in vasoactive factors. Among them, a reduction in the bioavailability of the vasodilator nitric oxide (NO) plays a pivotal role [7,8,9]. The importance of a NO imbalance in a kidney IR injury is highlighted in a number of experimental studies showing that maneuvers aimed to increase NO production/signaling have resulted in kidney protection against ischemic injury [10,11,12,13,14,15,16]. Indeed, NO administration to patients undergoing multiple valve replacement surgeries reduced post-operative AKI incidence and transition to chronic kidney disease stage 3 after one year of the surgery [17]. 

Nitric oxide production by the endothelial nitric oxide synthase (eNOS) is tightly regulated by multiple processes including: specific sub-cellular eNOS localization, eNOS interaction with other proteins, eNOS dimerization, and eNOS phosphorylation in multiple sites [18,19]. Moreover, endothelial nanomechanics modulate NO generation by an eNOS dependent mechanism: Soft endothelial cells release larger amounts of NO while the NO release is decreased when the endothelial cell stiffens (e.g., by rise in extracellular sodium and/or changes in cell shape) [20,21]. 

The epithelial sodium channel (ENaC) has a well described role in the kidney, lung, colon, and sweat glands epithelia where it maintains cellular sodium and water homeostasis [22]. ENaC is not only expressed in epithelial cells but can also be found in endothelial and smooth muscle cells [23,24,25,26]. In the endothelium, ENaC can modulate the cellular mechanics by controlling the cell softness or stiffness [27]. This is important since, as mentioned before, cortical stiffness of the endothelial cell is closely associated to NO generation [20,21]. Therefore, endothelial ENaC may modulate an endothelial function and a NO release. Evidence supporting a direct role of endothelial alpha-ENaC(αENaC) subunit in endothelial stiffness aroused from the observation that when αENaC expression or ENaC activity is increased in cultured human endothelial cells or in the mouse aorta, it results in stiff endothelial cells and reduced NO production [28]. In addition, pharmacological ENaC inhibition in rat mesenteric arteries increased NO synthesis and endothelium-dependent vasodilation, and this effect was linked with increased eNOS activation [29]. Moreover, endothelial αENaC knockout mice displayed soft endothelial cells and increased aortic eNOS phosphorylation at Ser1177 in the basal state [30].

Given the impact of endothelial ENaC on the regulation of cell mechanics and NO equilibrium, we hypothesized that ENaC function in the endothelium might be relevant to ischemic AKI, a condition known to be associated with NO imbalance and sustained hypo-perfusion. Therefore, we tested the effect of endothelial αENaC knockout on the severity of ischemic AKI. 

## 2. Results

### 2.1. The Endothelial αENaC Deficiency Reduces Ischemic AKI Severity

**Figure 1:**
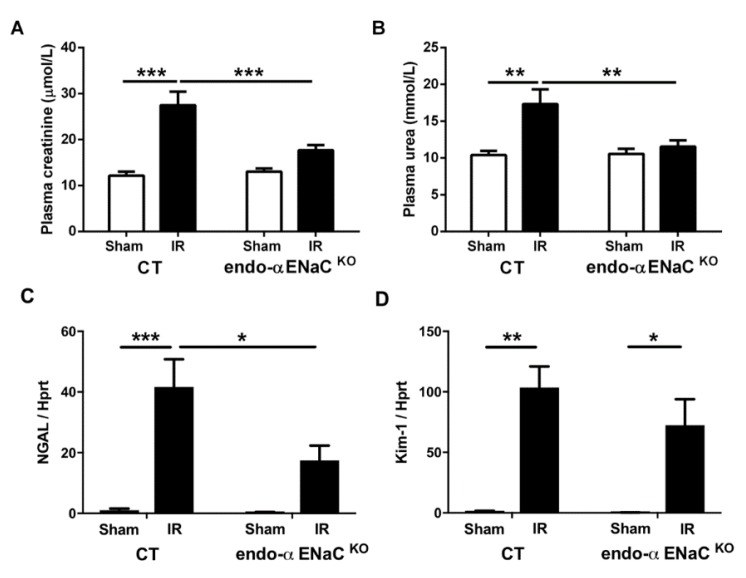
Endothelial sodium channel α (αENaC) deficiency protects against kidney injury induced by ischemia/reperfusion (IR). (**A**) Plasma creatinine and (**B**) plasma urea levels as indicators of the renal function. Whole kidney mRNA levels for (**C**) neutrophil gelatinase associated lipocalin-2 and (**D**) kidney injury molecule-1. Hypoxanthine guanine phosphoribosyltransferase (Hprt) expression was used as housekeeping gene for normalization. *n* = 8 per group. * *p* < 0.05, ** *p* < 0.01, and *** *p* < 0.001.

The effect of bilateral renal IR was tested in mice with αENaC deficiency in endothelial cells (endo-αENaC^KO^) and control littermates (CT). After 24 h of reperfusion, CT mice displayed renal dysfunction evidenced by a significant increase in plasma creatinine and urea. The absence of αENaC in the endothelium did not modify plasma creatinine and urea levels in sham mice and prevented renal dysfunction induced by the IR procedure (Figure 1A,B). The mRNA levels of neutrophil gelatinase associated lipocalin-2 (NGAL) and kidney injury molecule-1 (Kim-1), two sensitive biomarkers of kidney tubular injury, were highly augmented in CT IR mice. The increase of NGAL was significantly less pronounced in endo-αENaC^KO^ mice while the increase in Kim-1 was not affected (Figure 1C,D). 

**Figure 2:**
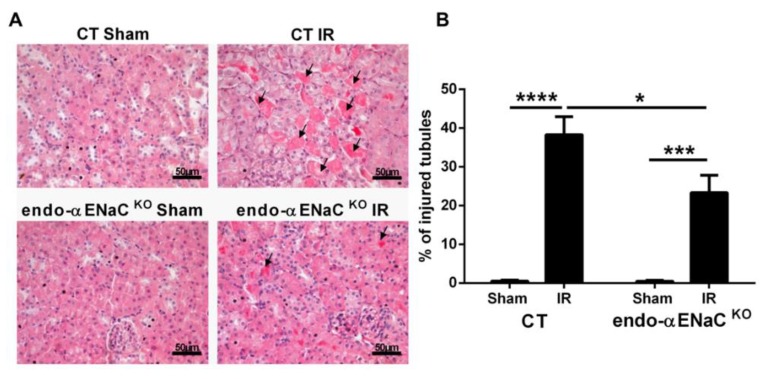
Endothelial αENaC deficiency reduces the tubular injury induced by IR**. (A**) Representative images from hematoxylin and eosin stained slides for the sham and IR groups in the control (CT) and mice with αENaC deficiency in the endothelial cells (endo-αENaC^KO^) after 24 h of reperfusion. The arrows indicate examples of injured tubules. (**B**) Quantification of the percentage of injured tubules presenting tubular casts, cell detachment, and/or tubular dilation. *n* = 8 per group. * *p* < 0.05, *** *p* < 0.001, and **** *p* < 0.0001.

Next, we analyzed the degree of tubular injury in hematoxylin-eosin stained slides. As expected, we found kidney IR induced tubular alterations such as cast formation, cell necrosis, and tubular dilation (Figure 2A). We noted that the extent of tissue injury was significantly reduced in the endo-αENaC^KO^ mice compared to the controls as depicted in the representative images (Figure 2A) and in the quantification of the percentage of injured tubules (Figure 2B). 

### 2.2. The Mice with αENaC Deficiency Recover Faster from the Hypoxia Induced by IR

**Figure 3:**
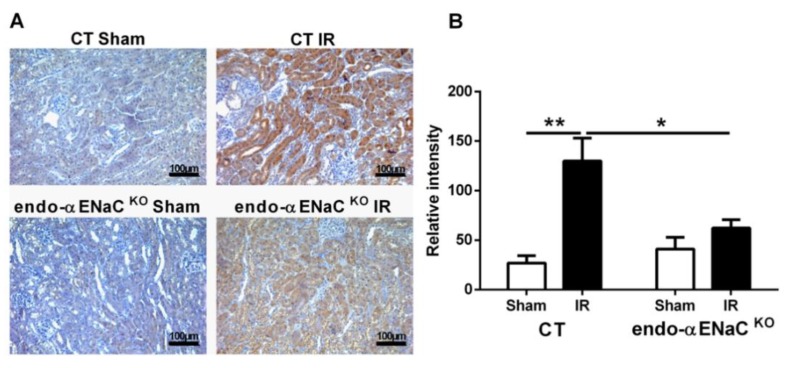
Endothelial αENaC deficiency promotes a faster recovery from hypoxia after IR**.** (**A**) Representative images from immuno-histochemistry against the pimonidazole adduct for sham and IR groups in control mice (CT) or mice with αENaC deficiency in the endothelial cells (endo-αENaC^KO^) after 2 h of reperfusion. (**B**) Quantification of the relative intensity staining for the pimonidazole adduct. *n* = 5 per group. * *p* < 0.05 and ** *p* < 0.01.

To test the hypothesis that endothelial αENaC deficiency might translate into increased NO production and therefore an improved perfusion of the tissue after an ischemic insult, we analyzed the hypoxia levels in the acute phase after reperfusion (two hours) by the Hypoxyprobe^TM^ method, as an indicator of the state of kidney hypoxia. In sham CT and endo-αENaC^KO^ mice, pimonidazole adduct as a marker of hypoxia was almost undetectable (Figure 3A). In contrast, renal IR induced intense pimonidazole staining (Figure 3A,B). The absence of αENaC in the endothelium was able to reduce the degree of hypoxia in the early phase after reperfusion as evidenced by the lower pimonidazole immunostaining intensity (Figure 3A,B).

### 2.3. Pharmacological ENaC Inhibition Promotes eNOS Activation in Human Endothelial Cells

**Figure 4:**
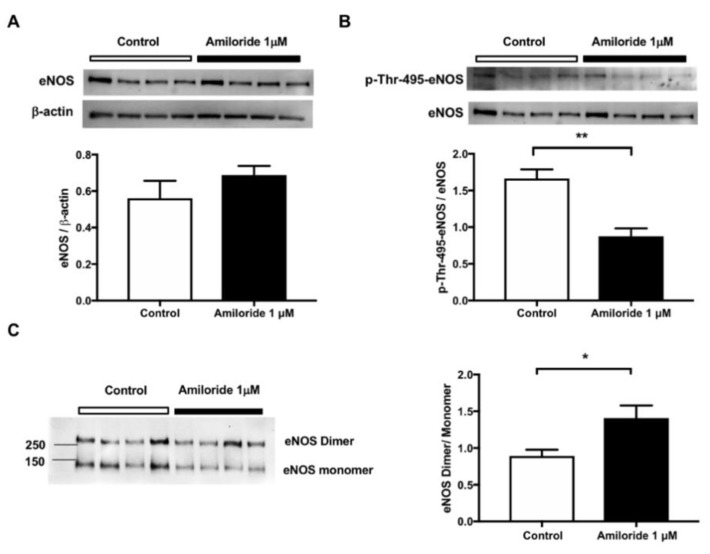
Pharmacological ENaC inhibition in human endothelial cells (HMEC-1) increases endothelial nitric oxide synthase (eNOS) activation/coupling**.** HMEC-1 cells were incubated with vehicle (control) or amiloride 1 μM for 24 h. The (**A**) eNOS protein and (**B**) eNOS phosphorylation at threonine 495 levels were determined by Western blot. (**C**) The dimer/monomer ratio of eNOS was determined in low-temperature/low-voltage SDS electrophoresis and Western blot. *n* = 4 per group, each lane represents an independent experiment for each condition. * *p* < 0.05 and ** *p* < 0.01.

The improved kidney perfusion recovery after IR in the endo-αENaC^KO^ mice suggests that increased eNOS activation in the endothelial cells might be responsible for this effect. Therefore, we evaluated the influence of ENaC inhibition on eNOS activation in human endothelial cells (HMEC-1). As shown in Figure 4A, acute ENaC inhibition with amiloride did not affect the eNOS protein expression levels. However, the phosphorylation of eNOS at the threonine 495 (which inactivates eNOS) was significantly reduced by ENaC inhibition (Figure 4B). Moreover, the eNOS dimer/monomer ratio, which reflects eNOS coupling and activation, was increased after ENaC inhibition with amiloride (Figure 4C). 

**Figure 5:**
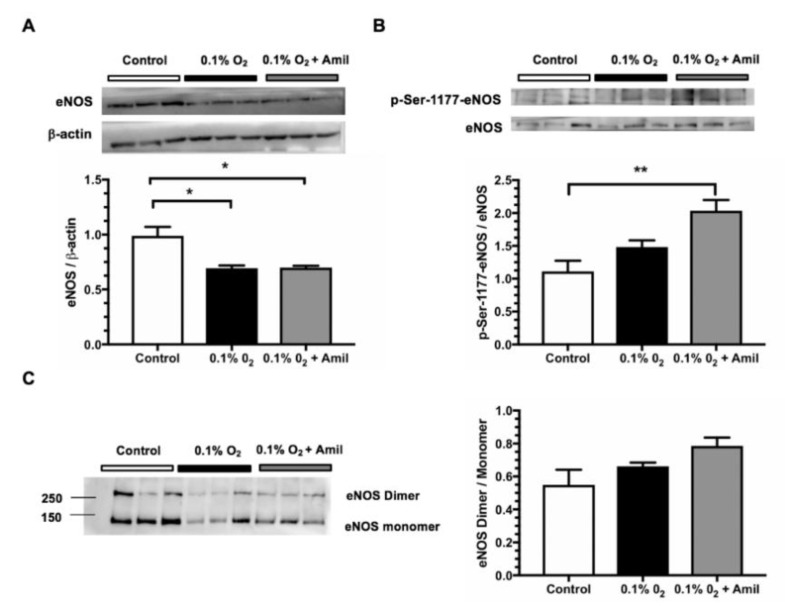
Effect of ENaC inhibition in human endothelial cells (HMEC-1) on eNOS activation after hypoxia/reoxygenation**.** HMEC-1 cells were incubated in normal oxygen concentration (21%) (control) or in hypoxic conditions (0.1% O_2_) with or without amiloride 1 μM addition for 24 h. The cells were returned to normal oxygen for 4 h reoxygenation and the proteins were extracted. (**A**) eNOS protein levels and (**B**) eNOS phosphorylation at Ser1177 determined by Western blot. (**C**) The dimer/monomer ratio of eNOS was determined in low-temperature/low-voltage SDS electrophoresis and Western blot. *n* = 3 per group, each lane represents an independent experiment for each condition. * *p* < 0.05 and ** *p* < 0.01.

To simulate the conditions that endothelial cells undergo during renal ischemia, we subjected the endothelial cells to hypoxia for 24 h (0.1% oxygen) and re-oxygenation (21% oxygen) for 4 h and tested if ENaC inhibition in hypoxic endothelial cells would still have a positive effect on eNOS activation. As shown in Figure 5A, the total eNOS protein levels were significantly reduced by hypoxia, an effect that was not prevented by ENaC inhibition. In contrast, when analyzing the proportion of eNOS phosphorylated at the Ser1177, we observed a significant increase in this phosphorylation in the cells in which ENaC was inhibited in hypoxic conditions (Figure 5B). The dimer/monomer ratio only trended to increase in the endothelial cells with amiloride treatment (Figure 5C). 

## 3. Discussion

In this study, we found that endothelial αENaC deficiency is associated with kidney protection against ischemic injury through a faster recovery from the prolonged hypoxia linked to persistent reduction in renal blood flow after an IR episode. This effect could be mediated by an increased vasodilatory or decreased vasoconstriction response of the renal vasculature upon IR in the mice with αENaC deficiency. We observed in cultured human endothelial cells that pharmacological ENaC inhibition induced with amiloride increased eNOS activation/coupling. This effect could explain a shift to vasodilation and a faster recovery after renal IR injury. In recent years, a role for endothelial ENaC in the regulation of vascular tone and NO production has been reported [22,31]. The endothelial ENaC regulates cell stiffness altering the synthesis and the release of NO. When ENaC expression is increased, the endothelial cell stiffs and the NO production is reduced [28]. In contrast, in the absence of ENaC the endothelial cell softens and the NO generation is facilitated; an effect that appears to be dependent on eNOS regulation by ENaC [20,22,28,30,32]. In mesenteric arteries, ENaC pharmacological inhibition increases eNOS phosphorylation (Ser1177) and endothelium dependent vasodilation [29]. Moreover, we previously demonstrated that the absence of endothelial αENaC expression led to a significant adaptation in the vessels and increased eNOS Ser1177 phosphorylation in the basal state, suggesting that ENaC inhibits eNOS dependent NO production. On the other hand, decreased endothelial αENaC expression/activity led to an increase of eNOS-dependent NO production [30]. Since NO is an essential factor for an adequate kidney perfusion and for the recovery after an ischemic AKI episode, we hypothesized that endothelial ENaC activity may be part of the vicious circle leading to sustained hypo-perfusion after renal IR. Indeed, the mice with αENaC deficiency in the endothelium presented significantly less tubular and functional injury after 24 h of the IR episode and less hypoxia in the early phase after reperfusion as compared to the control. Given the observations that in mouse vessels (aorta) αENaC deficiency induced an increase in eNOS activation and our results showing that ENaC inhibition in endothelial cells increased eNOS activation, the present data suggests that the kidney protective effect against ischemic AKI in the endo-αENaC^KO^ mice was mainly mediated by a better NO bioavailability, vasodilation, and improved perfusion. Supporting this hypothesis, it was recently shown that endothelial ENaC mediates the aldosterone-induced reduction in endothelial eNOS phosphorylation [33]. Moreover, in Dahl salt-sensitive rats, a high salt diet significantly increased plasma aldosterone and ENaC activity in endothelial cells; effects associated with blunted endothelium-dependent artery relaxation. In these rats, amiloride administration increased phosphorylated eNOS and NO production, and prevented the high salt-induced loss of vasorelaxation [34]. In addition, in Sprague-Dawley rats, reduced ENaC activity and expression improves endothelium-dependent artery relaxation [35]. On the other hand, it was recently shown that endothelial αENaC subunit deletion impairs the endothelium dependent vasodilation [36]. This discrepancy might be explained by the differential role that ENaC appears to have in conduit arteries where ENaC has a role as a vasoconstrictor as compared to resistance arteries in which ENaC participates in vasodilation [37].

The exact mechanisms by which endothelial ENaC deficiency/inhibition alters NO production remain unclear. Here, we report that ENaC inhibition promoted eNOS activation by reducing the phosphorylation of the Thr495 of eNOS and favoring the dimerization/coupling of eNOS. Another mechanism by which ENaC modulates NO synthesis is by modulating the L-arginine entry into the endothelial cell [24]. Guo et al. showed that increases of intracellular sodium concentration are associated with a reduction of cationic amino acid transporter-1 activity and therefore it is likely that increased ENaC activity affects endothelial cell function by this mechanism. Indeed, there was a reduction of NO production that was reversed by ENaC inhibition with amiloride or benzamil in HUVEC cells subjected to shear force to activate ENaC [24].

Our results highlight the contribution of endothelial αENaC in organ damage. In support of this, previous studies have shown that endothelial ENaC inhibition improves endothelial function, reduces aortic stiffening, and prevents left ventricular diastolic dysfunction in a mice model of Western diet induced obesity [38,39]. Moreover, inhibition of endothelial ENaC with amiloride also reduced coronary endothelium remodeling and permeability, decreased cardiac macrophage infiltration, and M1 inflammatory polarization [39]. In a model of aldosterone-induced endothelium stiffness and aortic dysfunction, it was demonstrated that endothelial αENaC subunit deletion induced a smaller aortic endoplasmic reticulum stress, increased eNOS activation, decreased endothelial permeability, and prevented the endothelium stiffness and aortic relaxation dysfunction [33].

Here, we show that endothelial αENaC deficiency was associated with amelioration of the acute deleterious effects of an ischemic episode in the kidney. Whether this protective effect could be translated into prevention of the AKI to chronic kidney disease transition remains to be explored. 

Altogether, our data highlight the impact of endothelial ENaC on eNOS regulation and endothelial function, which influences the response to a pathological situation such as ischemic AKI in which the endothelium and NO play an essential role for a faster recovery. 

## 4. Materials and Methods

### 4.1. Mouse Models

All experiments were conducted in accordance with the INSERM guidelines and the European legislation for the care and use of laboratory animals, approval number 4486 2016010616217743, 5 October 2016. The αENaC subunit (*Scnn1a*) knockout mice, called endo-αENaC ^KO^, were obtained by crossing αENaC^f/f^ floxed mice (kindly provided by Bernard Rossier, Lausanne, Switzerland [40]) with transgenic mice expressing Cre recombinase under the control of Tie2 promoter on C57Bl/6 genetic background (The Jackson Laboratory, Bar Harbor, ME, USA). αENaC ^f/f^ littermates lacking the Tie2-Cre transgene were used as controls. This model of endothelial αENaC deficiency was previously characterized and showed normal blood pressure, morphology, creatinine clearance, and renal sodium handling as compared to the control mice [30]. All the animals of this study were 10–12-week-old males housed in a temperature-controlled facility with a 12–12-h light/dark cycle and were allowed free access to food and water. 

### 4.2. Renal Ischemia/Reperfusion Model

Male mice were anesthetized with an intra-peritoneal injection of sodium pentobarbital (60 mg/kg). The mice were placed in a heating pad and the temperature was controlled at 37 °C through a rectal probe. The mice underwent bilateral flank incisions and both kidney pedicles were dissected. Renal ischemia was induced by placing vascular clamps over the renal pedicles during 22 min. After the ischemia period, the clamps were released and the mice were administered 1 mL of 0.9% NaCl (37 °C). The incisions were closed with 5–0 sutures and the reperfusion was allowed for 24 h. Sham animals underwent the same procedure without the clamping of the renal pedicle. After 24 h of the ischemia or sham procedure, a blood sample was taken by cardiac puncture, and plasma creatinine and urea concentrations were determined with an automatic analyzer (Konelab 20i, Thermoscientific, Waltham, MA, USA). The mice were killed and the kidneys were harvested. The left kidney was snap-frozen for molecular analyses and the right kidney was fixed in a Bouin fixative solution for histology studies. 

### 4.3. Gene Expression Analysis

Total RNA from the whole kidney was extracted using the TRIZOL^®^ reagent (Life Technologies, Carlsbad, CA, USA), according to manufacturer protocol. Reverse transcription of mRNA (1 μg) was performed using the Superscript II Reverse Transcriptase kit (Life Technologies). Transcript levels of genes were analyzed by real-time PCR (fluorescence detection of SYBR Green) in an iCycler iQ (Bio-Rad, Hercules, CA, USA). For each sample, mRNA levels were normalized to the housekeeping gene, HPRT. The sequences of the primers used were as follows: Kim-1; F 5′-TGTCGAGTGGAGATTCCTGGATGGT-3′, R 5′-GGTCTTCCTGTAGCTGTGGGCC-3′, NGAL; F 5′-GGACCAGGGCTGTCGCTACT-3′, R 5′-GGTGGCCACTTGCACATTGT-3′, HPRT; F 5′-TCTAACTTTAACTGGAAAGAATGTC-3′, R 5′-TCCTTTTCACCAGCAAGCT-3′/Eurogentec).

### 4.4. Kidney Histology and Tubular Injury Quantification

The fixed kidneys were then dehydrated and embedded in paraffin. Sections (4 μm) were cut and stained with hematoxylin and eosin. For each mouse, 10 subcortical fields were visualized and analyzed under a Leica DM4000 microscope at a magnification of 200×. The percentage of tubules displaying injury (cast formation, epithelial cell necrosis and detachment, and tubular dilation) was blindly analyzed.

### 4.5. Hypoxia Quantification

In another set of mice (*n* = 5 per group), hypoxia was detected by using the hypoxyprobe TM-1 Omni kit (Hypoxyprobe Incorporation, USA). The bilateral kidney ischemia was induced, and 2 h after the reperfusion the mice were injected with pimonidazole HCl. At 3 h after reperfusion the mice were killed and the kidneys were rapidly fixed by aortic perfusion of Bouin’s solution. Pimonidazole adducts were detected by immuno-histochemistry in 4 μm paraffin sections. The intensity of the signal was quantified by using the ImageJ software.

### 4.6. Human Endothelial Cell Culture and ENaC Inhibition

Human endothelial cells (HMEC-1, ATCC® CRL-3243^TM^) were kept in MCDB131 culture media (Gibco/BRL) supplemented with 10 mM glutamine (Gibco/BRL), 1 mg/mL hydrocortisone, 10 ng/mL Epidermal Growth Factor (EGF, Sigma), 10% fetal bovine serum, and antibiotics (100 U/mL penicillin; 100 μg/mL streptomycin; 0.25 μg/mL amphotericin). The cells were kept at 37 °C with a 100% relative humidity and 5% CO_2_. For the experiments the cells were used in passage 1–3 and were grown to an 80–90% confluency. The cells were incubated with water (control) or amiloride 1 μM for 24 h. Afterwards, the cells were harvested and lysed with an ice cold lysis buffer (50 mM Tris-HCl pH 8.0, 120 mM NaCl, 0.5% NP40, 100 mM NaF, 0.2 mM NaVO_3_, 1 μg/mL aprotinin, 1 mM PMSF, 1 μg/mL leupeptin) for 10 min to obtain the total protein. For the groups of cells that were exposed to hypoxic conditions, the vehicle or amiloride 1 μM was added, and the cells were put in a modular incubator chamber (Billups-rothenberg, Del Mar, CA, USA) with a nitrogen balance containing 0.1% oxygen and 5% CO_2_ for 24 h. After the incubation, the medium culture was replaced and the cells were allowed to recover for 4 h in a normal oxygen concentration (21%) before harvesting and lysis. 

### 4.7. Western Blot Analysis

The proteins obtained from the cell lysates (10 μg) were resolved on 8.5% SDS-PAGE gels and transferred to PVDF membranes. Primary antibodies were incubated overnight at 4 °C, and secondary antibodies were incubated for 90 minutes at room temperature. The membranes were stripped after detection of the phosphorylated eNOS in order to detect total eNOS levels. Antibodies and dilutions were used as follows: eNOS (BD Transduction Lab, Allschwil, Switzerland, 610297, 1:5000), p-Thr-495 eNOS (Cell signaling, Leiden, The Netherlands, 9574S, 1:1000), and β-actin-HRP (Abcam, Cambridge, UK, ab49900, 1:50,000). β-actin was used as a loading control. The amount of proteins was detected using a chemiluminiscence kit (Millipore, Burlington, MA, USA) in the ChemiDoc System (Bio-Rad). For the eNOS dimer and monomer quantification, non-boiled samples in reducing conditions and containing 20 μg of total protein were resolved in a 6% SDS-PAGE at 4 °C and low voltage (60 V). Then, the proteins were transferred to a PVDF membrane and Western blot analysis for eNOS was performed as described above. 

### 4.8. Statistical Analysis

The results are reported as mean ± standard error of mean (SEM). Data analysis was performed with GraphPad Prism (V7, GraphPad Software, San Diego, CA, USA). For comparison of more than two groups, a one-way ANOVA with Bonferroni post-tests was used. For the cell culture experiments where a two-group comparison is made a Student *t*-test was performed. *p* values <0.05 were considered statistically significant. Asterisks refer to: * *p* < 0.05; ** *p* < 0.01; *** *p* < 0.001; **** *p* < 0.0001. 

## Abbreviations

ENaC	Epithelial sodium channel
αENaC	Epithelial sodium channel alpha subunit
NO	Nitric oxide
eNOS	Endothelial nitric oxide synthase
IR	Ischemia / reperfusion
Kim-1	Kidney injury molecule-1
NGAL	Neutrophil gelatinase associated lipocalin
Hsp90	Heat shock protein 90
AKI	Acute kidney injury
CKD	Chronic kidney disease
CT	Control littermates
endoαENaC^KO^	Epithelial sodium channel alpha subunit knockout in endothelial cells
